# Supplementary material for: FEAST: A flow cytometry-based toolkit for interrogating microglial engulfment of synaptic and myelin proteins
Source: Nat Commun. 2023 Sep 27;14:6015. doi: 10.1038/s41467-023-41448-7 (PMC10533836; doi:10.1038/s41467-023-41448-7)
Supplement: Supplementary file 1 — Supplementary Information [file 41467_2023_41448_MOESM1_ESM.pdf]

Supplementary Information:

FEAST: A flow cytometry-based toolkit for interrogating microglial engulfment of synaptic and myelin proteins

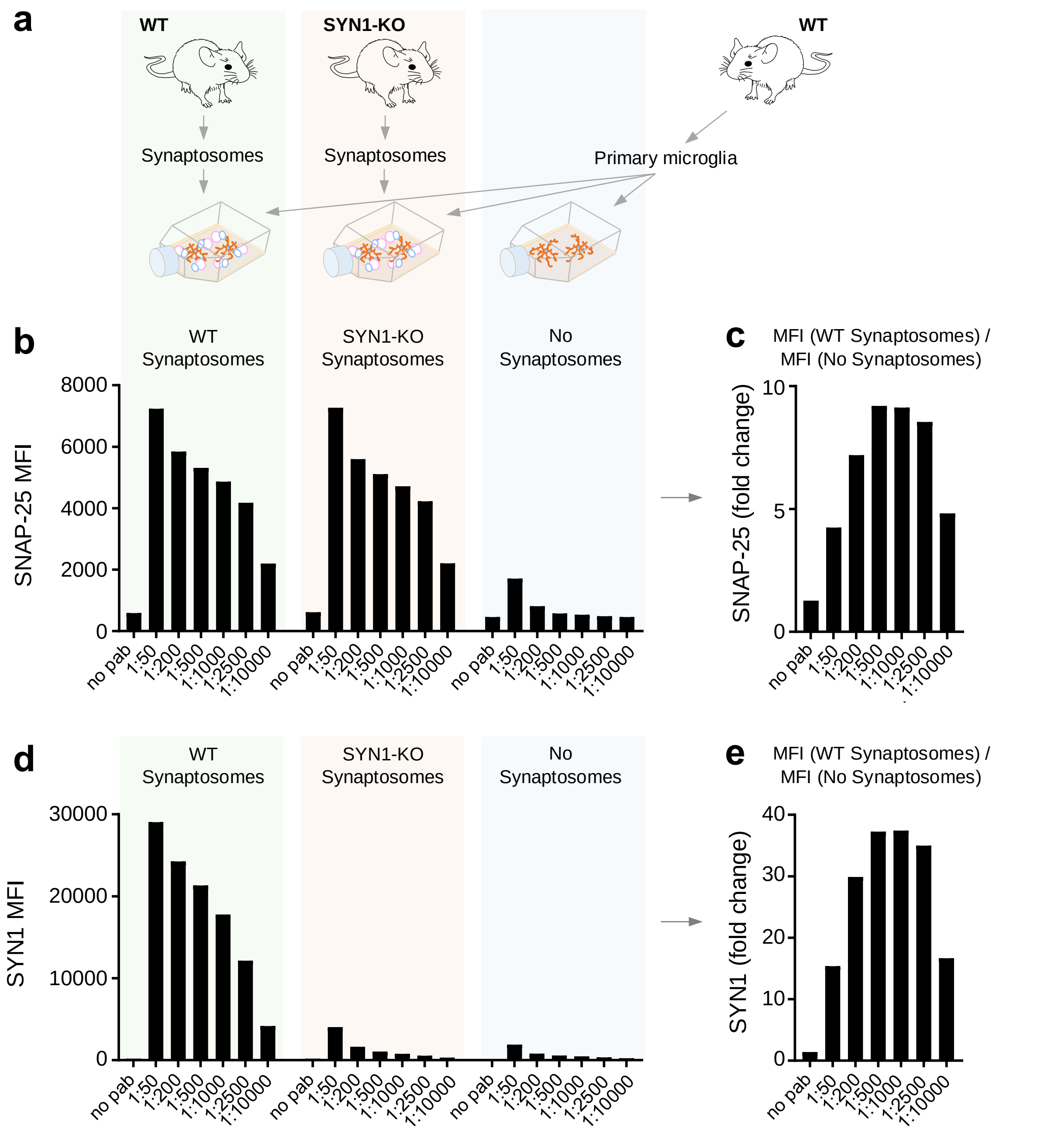

Supplementary Figure 1: Verification of hits from synaptic antibody screen in primary microglial cultures.
a) Diagram of the experimental setup for further validation of the anti-SNAP-25 and anti-SYN1 antibodies. Primary microglial cultures were generated from WT mice by Dounce homogenization followed by 40% Percoll enrichment and split into three different groups. One group was treated with synaptosomes from WT mice, the second group was

treated with synaptosomes from SYN1-KO mice, and the third group was left untreated. The cells from each group were harvested, fixed, split into 7 samples, permeabilized, and stained intracellularly with no primary antibody (no pab) or different concentration of anti-SNAP-25-AF594 and anti-SYN1-AF647.

b) MFI of SNAP-25 staining in cells treated with synaptosomes from WT and SYN1-KO mice.

c) Fold change of the SNAP25 MFI between cells treated with synaptosomes and untreated cells indicate an optimal concentration range of 1:500-1:1000.

d) MFI of SYN1 staining in cells treated with synaptosomes from WT and SYN1-KO mice.

e) The fold change of the SYN1 MFI between cells treated with synaptosomes and untreated cells indicate an optimal concentration range of 1:500-1:1000.

Source data provided.

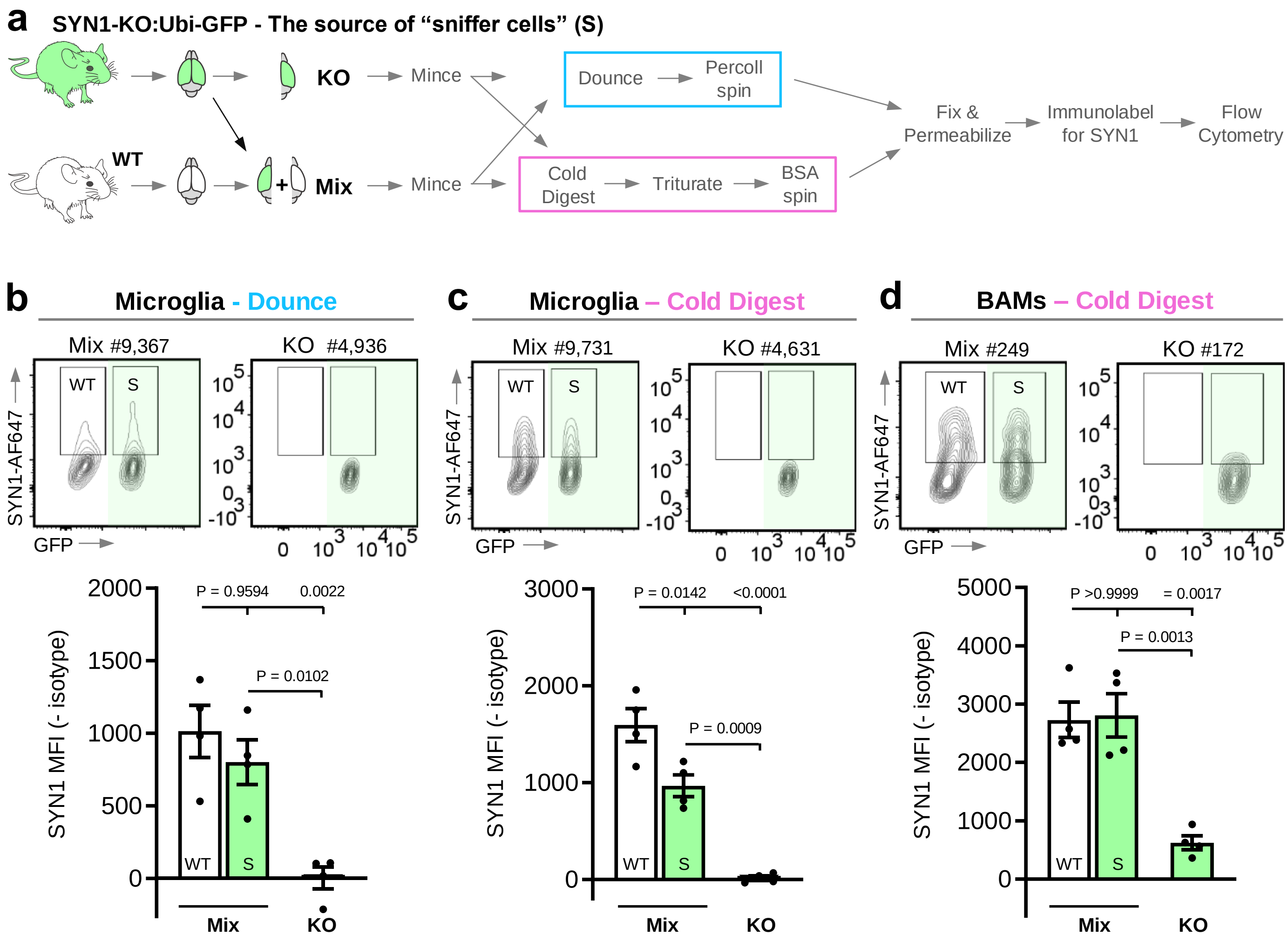

Supplementary Figure 2: Assessment of false positive signal in microglia and BAMs following Dounce homogenization or cold enzymatic digestion using "sniffer cells."

a) Schematic of the experimental design for evaluation of false-positive signal using "sniffer cells". "Sniffer cells" for false SYN1 signal were introduced by mixing brain tissue from SYN1-KO mice (crossed with Ubi-GFP mice for identification) with brain tissue from WT mice. After mincing, the tissues were either mechanically dissociated by Dounce homogenization or enzymatically digested using *Bacillus licheniformis* protease at 4 °C (Cold Digest) followed by trituration. All samples were then processed for flow cytometric analysis of SYN1.

b) Microglia isolated by Dounce homogenization. Representative contour plots showing SYN1 signal for WT and "sniffer cells" (S) in the mixed sample (separated on the x-axis based on GFP expression) as well as the KO sample (only GFP+ microglia). The gates for SYN1^{+} cells are based on the fluorescence of their respective isotype controls (<1% cells in positive gate). The mean fluorescent intensity (MFI) for SYN1 with isotype controls subtracted is depicted for all microglia. Note, Dounce homogenization does not yield sufficient of BAMs for analysis. ~10,000 microglia per sample.

c) Microglia isolated by cold digestion. The data are displayed similarly to microglia isolated by Dounce homogenization in panel b.

d) BAMs isolated by cold digestion. The data are displayed similarly to microglia in panel c.

b-d) Cells were gated on live, single, CD45^{+}, CD11b^{+}, CD64^{+}, and GR1^{-} and microglia were further gated on P2Y12^{high} while BAMS were gated on CD38^{high}. CX3CR1 and CD206 were not used as they were found to be affected by incubation with *Bacillus licheniformis* protease. n = 4 mice (2 females and 2 males). Error bars indicate the standard error of the mean. Statistical analysis: One-way ANOVA, Bonferroni's multiple comparisons test. Source data provided.

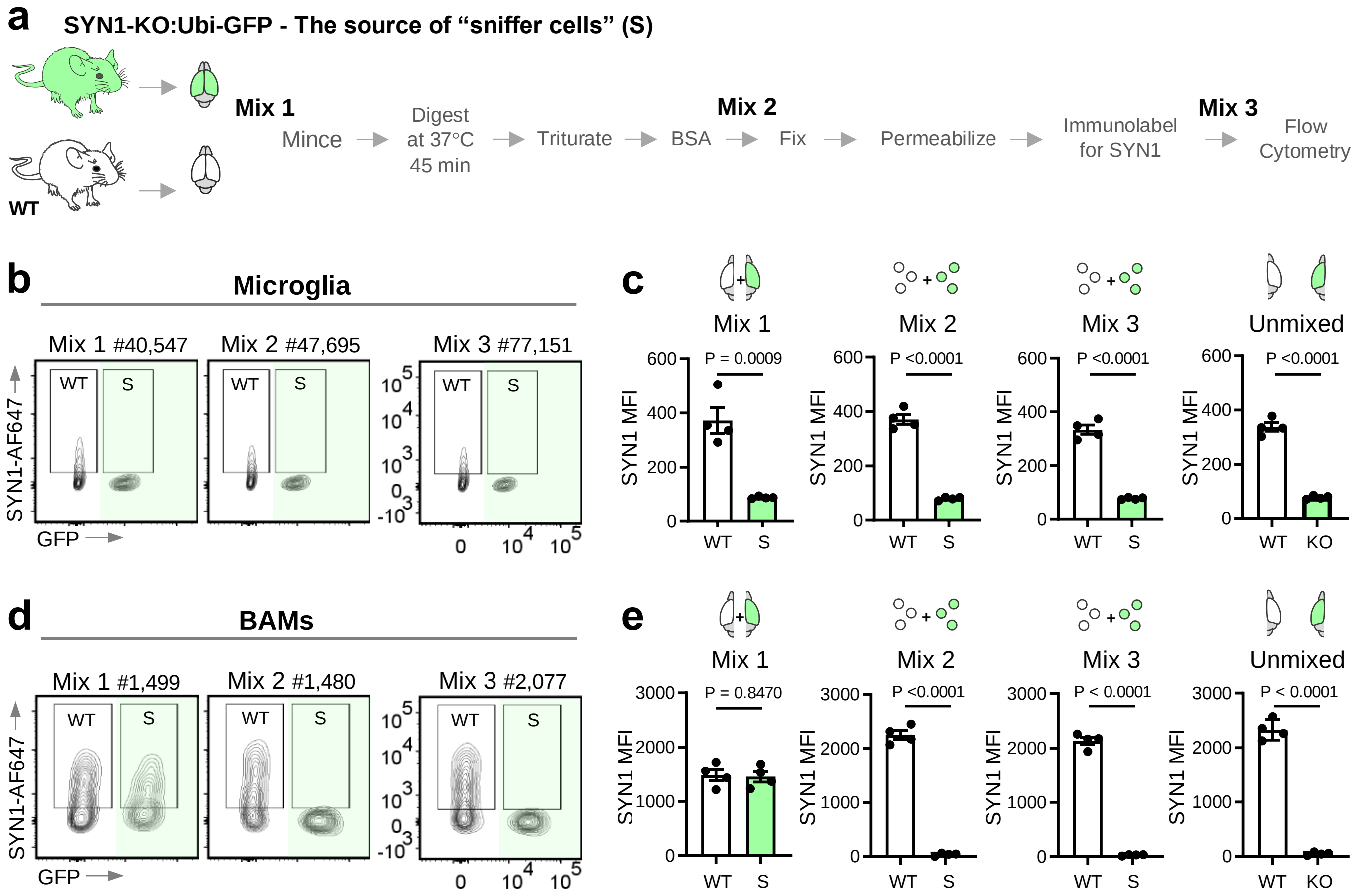

Supplementary Figure 3: Assessment of which isolation steps generate false positive signal in microglia and BAMs.

a) Schematic of the experimental design for evaluation of false-positive signal using "sniffer cells". "Sniffer cells" for identification of false SYN1 signal were introduced at 3 different steps of the procedure. Before mincing "Mix 1", before fixation of the single cell suspension "Mix 2", and just before examining the samples on the flow cytometer "Mix 3". All samples were prepared by enzymatic digestion (Col-IV) at 37 °C and processed for flow cytometric analysis of SYN1.

b) Contour plots depicting SYN1 signal for WT and "sniffer cell" (S) microglia (separated on the x-axis based on GFP expression) for Mix 1, 2, and 3. Gates for SYN1^{+} cells are based on the fluorescence of their respective KO controls (<1% microglia in positive gate) that were not mixed with WT sample.

c) Mean fluorescent intensity (MFI) of SYN1 for all microglia in each of the mixed samples as well as "unmixed" samples consisting of WT or SYN1-KO tissue.

d) Contour plots depicting SYN1 signal for WT and "sniffer cell" (S) BAMs for Mix 1, 2, and 3. Gates for SYN1^{+} cells are based on the fluorescence of their respective KO controls (<1% BAMs in positive gate) that were not mixed with WT sample.

e) Mean fluorescent intensity (MFI) of SYN1 for all BAMs in each of the mixed samples as well as "unmixed" samples consisting of WT or SYN1-KO tissue.

b-e) Cells were gated on live, single, CD45^{+}, CD11b^{+}, CD64^{+}, and GR1^{-} and microglia were further gated on CX3CR1^{high} and P2Y12^{high} while BAMS were gated on CD206^{high} and CD38^{high}. n = 4 mice (2 females and 2 males). Error bars indicate standard error of the mean. Statistical analysis: unpaired two-tailed t-test. Source data provided.

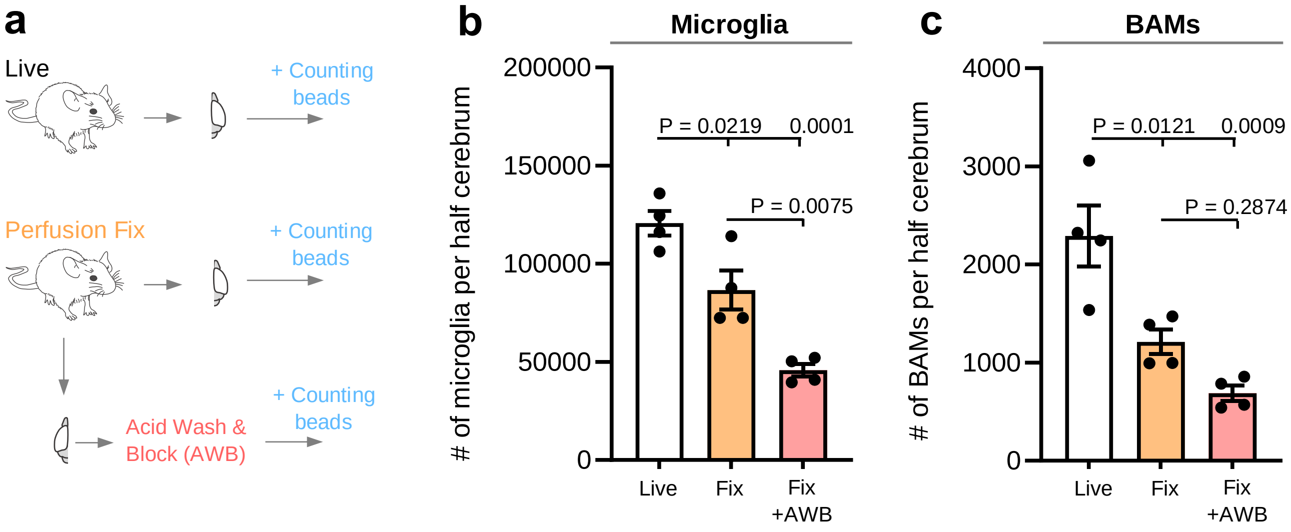

Supplementary Figure 4: Yield of cells harvested from perfusion-fixed tissue compared to live tissue.
a) Experimental schematic. Cells from half cerebra were harvested from live, HBSS-perfused tissue or perfusion-fixed tissue. Half of the cells harvested from perfusion-fixed tissue were further subjected to acid wash. Counting beads were added to each sample prior to the flow cytometric assessment for equal sample acquisition.
b) Number of microglia harvested from live tissue, perfusion-fixed tissue (without acid wash), and perfusion-fixed tissue + acid wash and blocking (AWB).
c) Number of BAMs harvested from live tissue, perfusion-fixed tissue (without acid wash), and perfusion-fixed tissue + AWB.
b-c) Cells were gated on DAPI (to identify nucleated cells), single cells, CD45^{+}, CD68^{+}, GR1^{-} and microglia were further gated on CX3CR1^{high} and P2Y12^{high} while BAMS were gated on CD206^{high} and CD38^{high}. n = 4 mice, all females. Error bars indicate standard error of the mean. Statistical analysis: One-way ANOVA, Bonferroni's multiple comparisons test. The SYN1 data for these experiments are presented in Fig 5b-c. Source data provided.

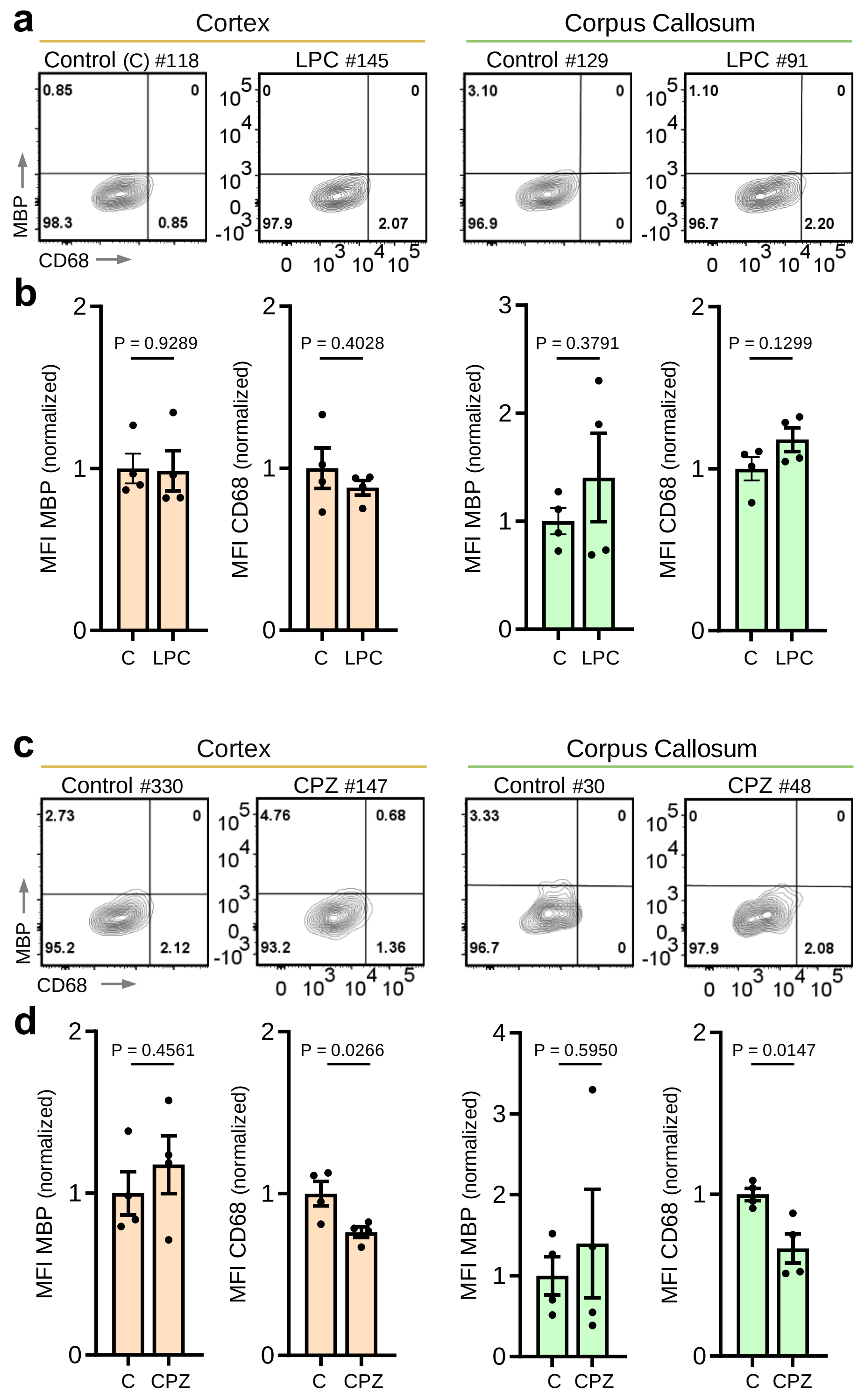

Supplementary Figure 5: BAMs do not engulf myelin following focal or global demyelination.

a) BAMs were harvested from perfusion-fixed tissue 7 days after injection of lysolecithin (LPC). Flow cytometry plots display MBP and CD68 signal in BAMs for both the cortex and the corpus callosum in control (non-injected) and LPC-injected mice.

b) MFI of MBP and CD68 signal in all microglia from cortex or corpus callosum (normalized to control).

c) BAMs were harvested from perfusion-fixed tissue after 3 weeks of cuprizone (CPZ) treatment. Flow cytometry plots display MBP and CD68 signal in all BAMs from cortex or corpus callosum in control (fed control diet) and CPZ-treated mice.

d) MFI of MBP and CD68 signal in all microglia from cortex or corpus callosum (normalized to control).

a-d) BAMs were gated on DAPI (to identify nucleated cells), single cells, $CD45^{+}$, $CD68^{+}$, $GR1^{-}$, $CX3CR1^{low}$, $P2Y12^{negative}$, $CD206^{high}$, and $CD38^{high}$, n = 4 mice per condition (2 females and 2 males). Error bars indicate standard error of the mean. Statistical analysis: unpaired two-tailed t-test. The microglia data for these experiments are presented in Fig 7. Source data provided.

a Gating strategy for live surface-stained microglia and BAMs

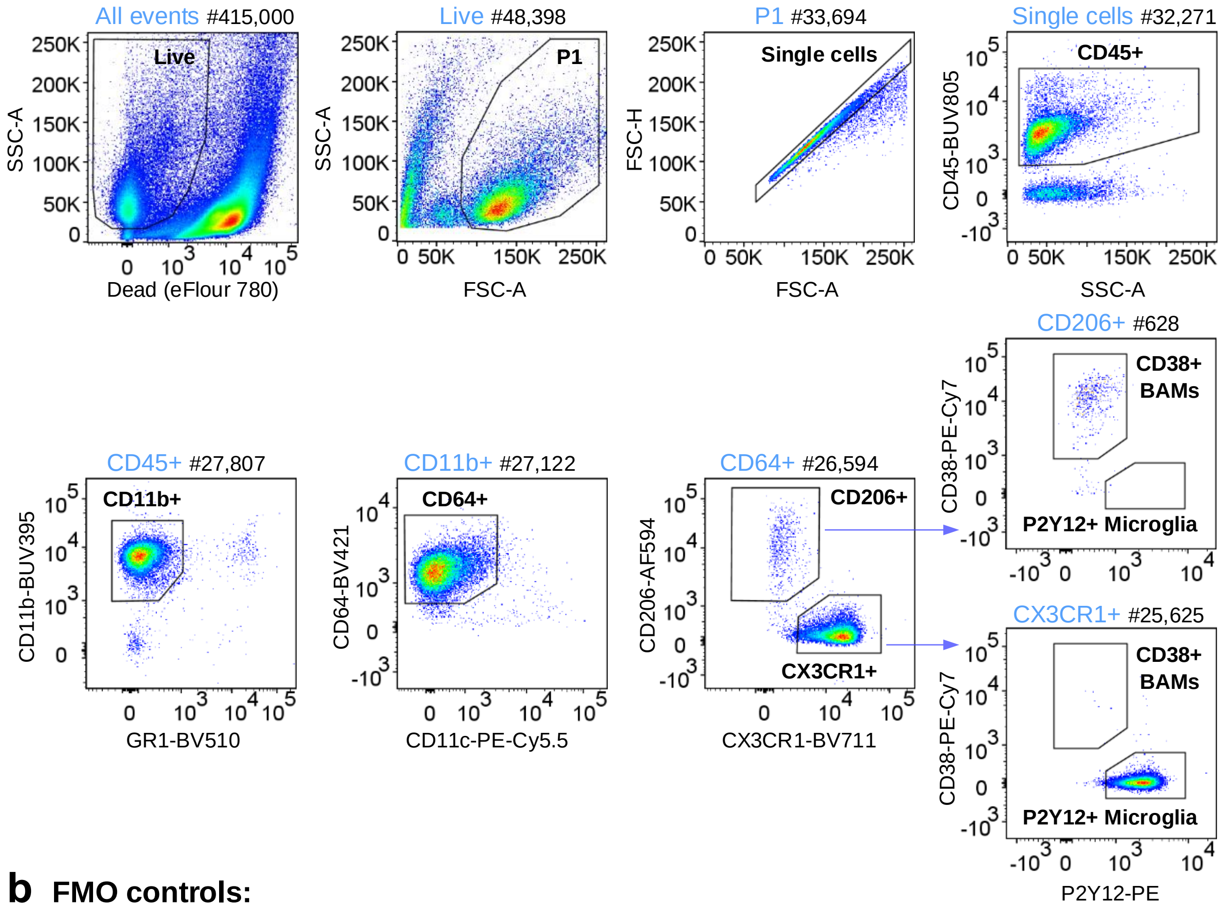

b FMO controls:

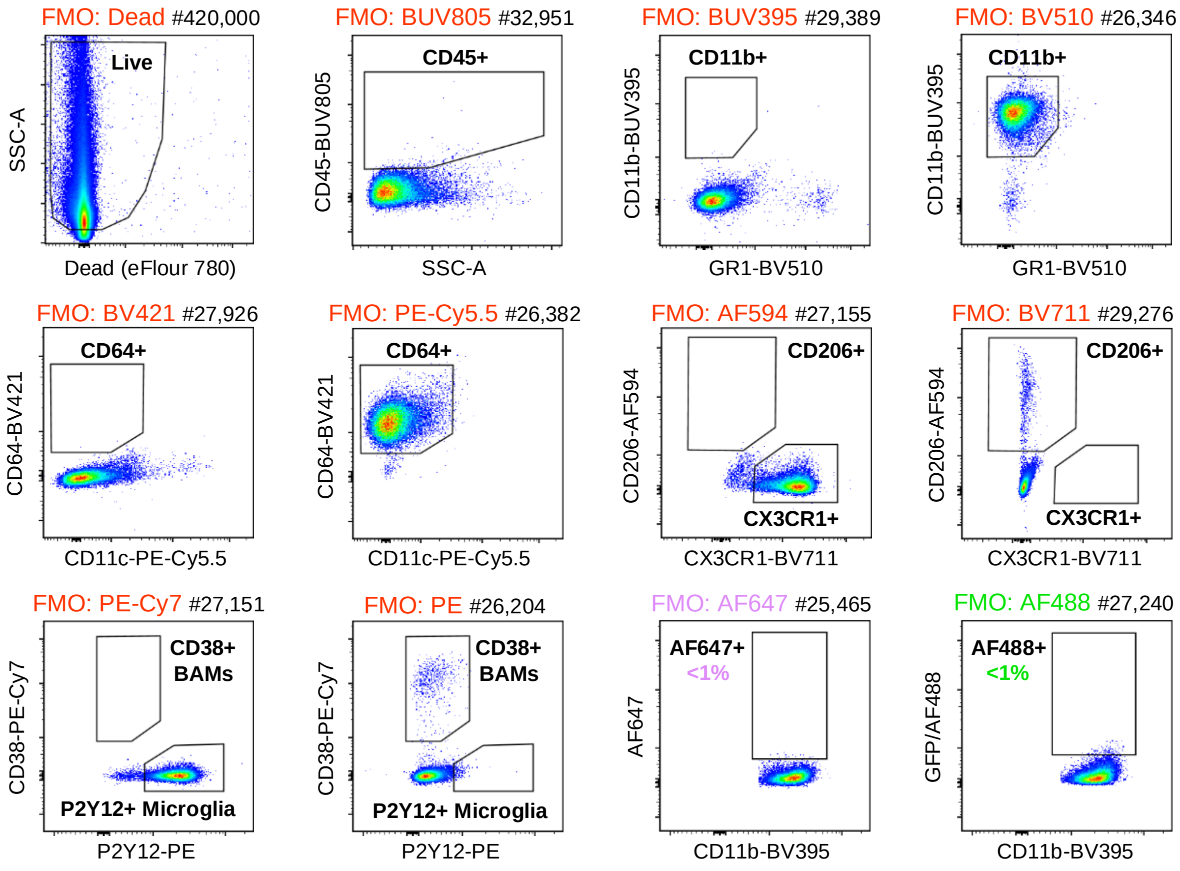

Supplementary Figure 6: 12-color flow cytometry antibody panel to identify microglia and quantify engulfment.
a) Eight step gating strategy to identify microglia and quantify engulfment in AF647. AF488/GFP was used to identify 'sniffer cells' or CTB-AF488. Cells were gated to remove dead cells (positive for Viability eFlour780). Single cells were determined based on SSC-A/FSC-A and FSC-H/FSC-A. Next, immune cells were gated based on CD45 expression and macrophages were identified based on the expression of CD11b and CD64 and absence of GR1 and CD11c (monocytes/granulocytes and dendritic cells). High P2Y12 and CX3CR1 expression and absence of CD206 and CD38 was used to identify microglia. High CD206 and CD38 expression, intermediate expression of CX3CR1, and absence P2Y12 was used to identify BAMs.
b) Display of the FMO (fluorescence minus one) controls for each of the twelve colors that guided the placement of the gates. The FMOs also served to verify the compensation settings.
See Supplementary Table 2 for antibody details.

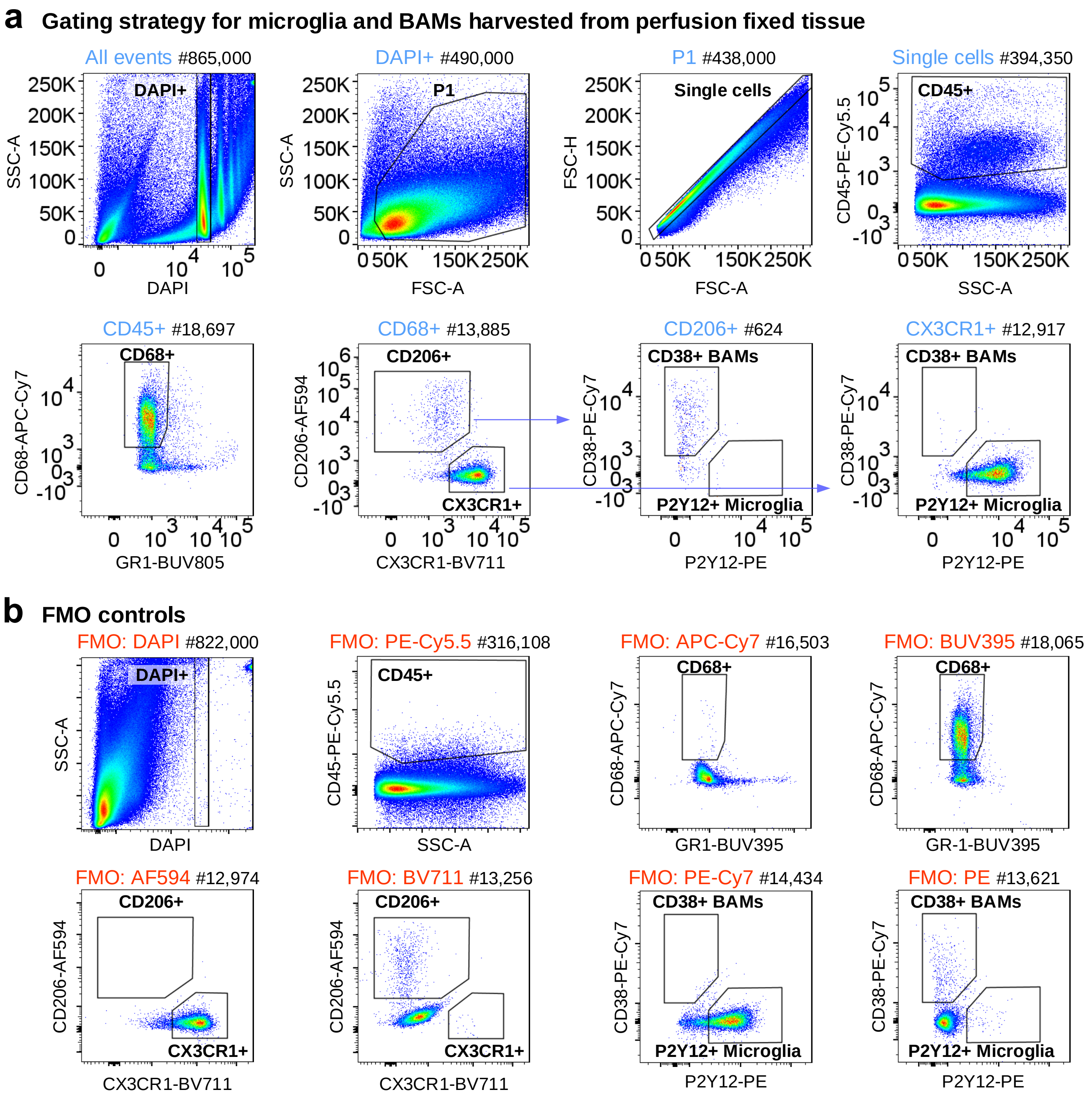

Supplementary Figure 7: Gating strategy for identifying microglia and BAMs harvested from perfusion-fixed tissue.

a) Nucleated cells were first distinguished from debris by DAPI. Single cells were then determined based on SSC-A/FSC-A and FSC-H/FSC-A. Next immune cells were gated based on CD45 expression and myeloid cells were identified based on the expression of CD68 and absence of GR1 (the antibodies against CD11b and CD64 used to identify live macrophages do not work well on fixed cells). High P2Y12 and CX3CR1 expression and absence of CD206 and CD38 were used to identify microglia. High CD206 and CD38 expression, intermediate expression of CX3CR1, and absence P2Y12 was used to identify BAMs.

b) Display of the FMO (fluorescence minus one) controls for each of the colors, which were used to guide the placement of the gates. The FMOs also served to verify the compensation settings.

See Supplementary Table 3 for antibody details.

a **Gating strategy used to identify microglia for Figure 1 (engulfment of AF488 and AF594)**

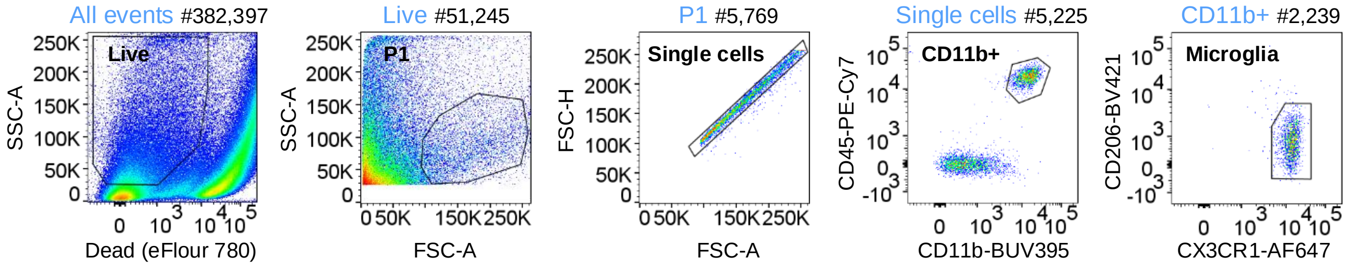

b **Gating strategy used to identify microglia for Figure 2a (engulfment of ZsGreen)**

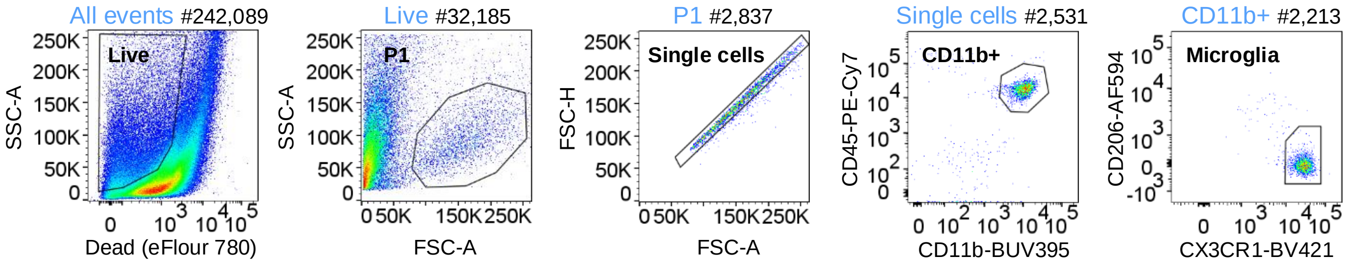

c **Gating strategy used to identify microglia for Figure 2b (engulfment of TdTomato)**

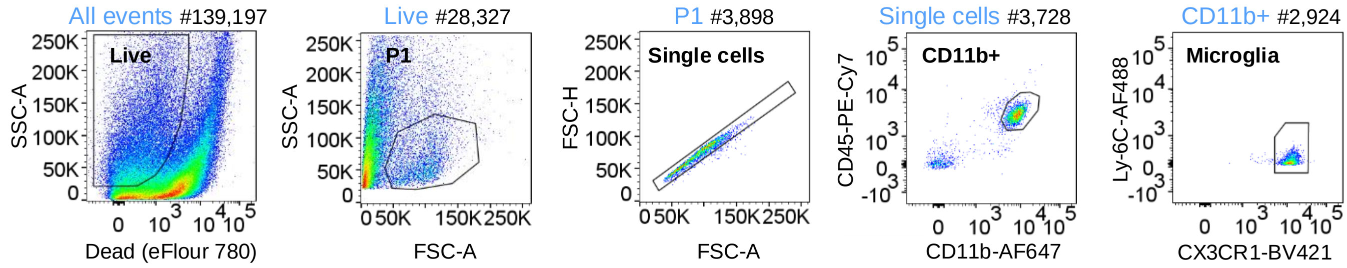

d **Gating strategy used to identify microglia for Figure 2c (engulfment of EGFP)**

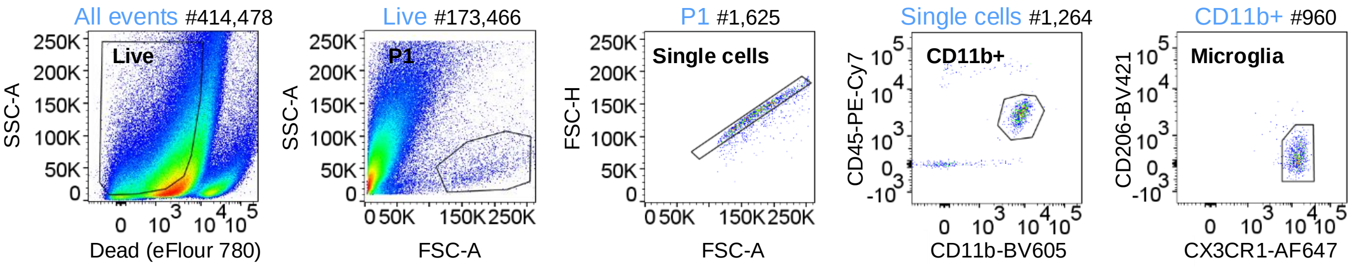

e **Gating strategy used to identify microglia for Figure 2d (engulfment of EYFP)**

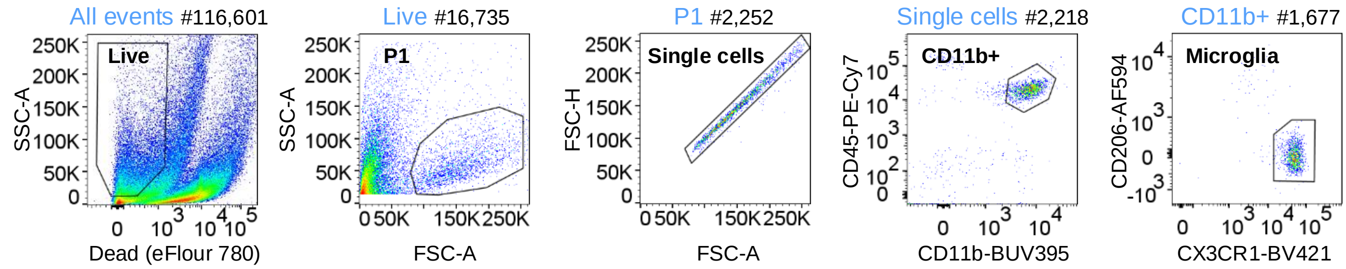

Supplementary Figure 8: Representative gating strategy used for Figures 1 and 2.
A five-step gating strategy was applied to identify live microglia for analysis of the engulfment of fluorescently-labeled synaptic terminals. Cells were gated to remove dead cells (positive for Viability eFlour780). Single cells were determined based on SSC-A/FSC-A and FSC-H/FSC-A and microglia were identified by gating $CD45^{int}$ $CD11b^{high}$ cells and then on cells expressing low CD206 or no Ly-6C and high CX3CR1.

Supplementary Tables

Antibody / dye	Species/isotype	Clone	Company	Cat#	Final conc.	Dilution
**Pre-synaptic**						
SNAP25-AF647 *	Ms IgG1	SMI81	Biolegend	836312	1 μg/ml	1:500
SYP Rb *	Rb mAb	SP11	Abcam	AB16659	~0.15 μg/ml	1:200
SYN1/2 Rb	Rb polyAb	N/A	Synaptic Systems	106003	2 μg/ml	1:500
SYN1 Rb (1)	Rb mAb	D12G5	Cell signaling	5297	0.4 μg/ml	1:200
SYN2 Rb	Rb mAb	D6S9C	Cell signaling	85852	0.05 μg/ml	1:400
SYN1 Rb (2)	Rb polyAb	N/A	Synaptic Systems	106 103	2 μg/ml	1:500
SYT Rb (1)	Rb polyAb	N/A	Synaptic Systems	105002	5 μg/ml	1:200
Piccolo GP	GP polyAb	N/A	Synaptic Systems	142 104	N/A (serum)	1:500
SYT Rb (2)	Rb polyAb	N/A	Cell signaling	3347	0.5 μg/ml	1:100
SYT Rb (3)	Rb mAb	D33B7	Cell signaling	14558	0.7 μg/ml	1:50
SYN1/2 Ck	Ck IgY	N/A	Synaptic Systems	106 006	N/A	1:500
Piccolo Rb	Rb polyAb	N/A	Synaptic Systems	142002	N/A (serum)	1:500
Bassoon Rb (1)	Rb polyAb	N/A	Synaptic Systems	141002	N/A (serum)	1:1000
Bassoon GP	GP polyAb	N/A	Synaptic Systems	141 004	N/A (serum)	1:500
Bassoon Rb (2)	Rb mAb	D63B6	Cell signaling	6897	2.4 μg/ml	1:200
SYT Ck	Ck polyAb	N/A	Aves	STG	0.8 μg/ml	1:500
**Excitatory**						
vGlut1 Rb *	Rb polyAb	N/A	Synaptic Systems	135303	2 μg/ml	1:500
vGlut1 GP	GP polyAb	N/A	EMD Millipore,	AB5905	N/A (serum)	1:1000
vGlut1 Ck	CK polyAb	N/A	Synaptic Systems	135 316	N/A	1:500
vGLut2 GP	GP polyAb	N/A	EMD Millipore,	AB2251	N/A (serum)	1:1000
vGlut2 Ck	Ck polyAb	N/A	Synaptic Systems	135 416	1 μg/ml	1:1000
vGlut2 Rb (1)	Rb mAb	D7D2H	Cell signaling	71555	0.8 μg/ml	1:50
vGlut2 Rb (2)	Rb polyAb	N/A	Synaptic Systems	135 403	2 μg/ml	1:500
**Inhibitory**						
VGAT Rb (1)	Rb polyAb	N/A	Synaptic Systems	131 003	1 μg/ml	1:1000
VGAT Rb (2)	Rb polyAb	N/A	Synaptic Systems	131 002	N/A (serum)	1:500
VGAT Ck	Ck polyAb	N/A	Synaptic Systems	131 006	2 μg/ml	1:500
VGAT GP (2)	GP polyAb	N/A	Synaptic Systems	131 004	N/A (serum)	1:500
GAD65 GP	GP polyAb	N/A	Synaptic Systems	198 104	N/A (serum)	1:500
VGAT GP (1)	GP polyAb	N/A	EMD Millipore	AB5062P	2 μg/ml	1:500
**Post-synaptic**						
GluA2/3 Rb	Rb polyAb	N/A	Abcam	Ab6438	N/A	1:500
Homer 1 Rb	Rb polyAb	N/A	Synaptic Systems	160 003	2 μg/ml	1:500
PSD95 Rb (2)	Rb mAb	D27E11	Cell signaling	3450	0.1 μg/ml	1:200
GluA1 Rb (1)	Rb polyAb	N/A	Abcam	Ab31232	1 μg/ml	1:1000
Homer 1b/c Rb	Rb polyAb	N/A	Synaptic Systems	160 023	5 μg/ml	1:200
PSD95 Rb (4)	Rb mAb	D74D3	Cell signaling	3409T	1 μg/ml	1:400
GluA4 Rb	Rb mAb	D41A110	Cell signaling	8070T	0.5 μg/ml	1:400
Homer 2 Rb	Rb polyAb	N/A	Synaptic Systems	160 302	2 μg/ml	1:500
PSD95 Rb (1)	Rb polyAb	N/A	Life Technology	51-6900	1.25 μg/ml	1:200
GluA1 Rb (3)	Rb polyAb	N/A	Millipore	ABN241	2 μg/ml	1:500
Homer 1 Ck	Ck polyAb	N/A	Synaptic Systems	160 006	1 μg/ml	1:1000
GluA1 Rb (2)	Rb mAb	D4N9V	Cell signaling	13185	0.5 μg/ml	1:200
PSD95 Rb (3)	Rb polyAb	N/A	Cell signaling	2507	0.6 μg/ml	1:100
**Control** (anti-synaptic)						
IgG-AF647	Ms IgG1	MOPC-21	Biolegend	400136	1 μg/ml	1:500
Ck-IgY	Ck IgY	N/A	Aves	N-1010	2 μg/ml	1:5,000
Rb Serum	Rb polyAb	N/A	Millipore Sigma	R9133	N/A	1:100
Rb IgG	Rb polyAb	Poly29108	Biolegend	910801	5 μg/ml	1:200
GP Serum	GP polyAb	N/A	Millipore Sigma	G9774	N/A	1:500

**Secondary**						
Goat-anti-Rb-IgG	PolyAb-AF647	N/A	Thermo Fisher	A-21245	1 μg/ml	1:2,000
Goat-anti-GP-IgG	PolyAb-AF647	N/A	Thermo Fisher	A-21450	1 μg/ml	1:2,000
Goat-anti-Ck-IgY	PolyAb-AF647	N/A	Thermo Fisher	A-21449	1 μg/ml	1:2,000
**Anti-MBP**						
P82H9-AF647 *	Ms IgG1	P82H9	Biolegend	850910	2.5 μg/ml	1:200
SMI 99-AF647 *	Ms IgG2b	SMI 99	Biolegend	808408	2.5 μg/ml	1:200
2H9-AF647 *	Ms IgG1	2H9	Novus Biologicals	22121	2.5 μg/ml	1:200
SMI 94-AF488	Ms IgG1	SMI 94	Biolegend	836506	2.5 μg/ml	1:200
**Controls** (anti-MBP)						
IgG1-AF488	Ms IgG1	MOPC-21	Biolegend	400134	2.5 μg/ml	1:200
IgG1-AF647	Ms IgG1	MOPC-21	Biolegend	400155	2.5 μg/ml	1:200
IgG2b-AF647	Ms IgG2b	MPC-11	Biolegend	400330	2.5 μg/ml	1:200

Supplementary Table 1: Antibodies used for *in vitro* screen
Antibodies recommended for FEAST are indicated in yellow and with an asterisk. These antibodies produced a significant increase in synaptic protein or myelin signal in cells fed the respective substrates compared to unfed controls. Chicken (Ck), Guinea Pig (GP), Mouse (Ms), and Rabbit (Rb). Monoclonal antibody (mAb) and polyclonal antibody (polyAb)

Antibody / dye	Species /isotype	Clone	Company	Conjuga-tion	Cat#	Conc.	Dilution
Viability Dye	N/A	N/A	Thermo Fisher	eFluor780	50-169-66	N/A	1:1,000
Anti-CD16/CD32	Rt IgG2b	2.4G2	BD Bioscience	None	553141	10 μg/ml	1:50
**Surface**							
Anti-CD45	Rt IgG2b	30-F11	BD Bioscience	BUV805	748370	1 μg/ml	1:200
Anti-CD11b	Rt IgG2b	M1/70	BD Bioscience	BUV395	563553	1 μg/ml	1:200
Anti-Gr1	Rt IgG2b	RB6-8C5	Biolegend	BV510	108438	2 μg/ml	1:100
Anti-CD64	Ms IgG1	X54-5/7.1	Biolegend	BV421	139309	5 μg/ml	1:40
Anti-CD11c	Ha IgG	N418	Thermo Fisher	PE-Cy5.5	35-0114-82	1 μg/ml	1:200
Anti-CD206	Rt IgG2a	C068C2	Biolegend	AF594	141712	2.5 μg/ml	1:100
Anti-CX3CR1	Ms IgG2a	SAD011F11	Biolegend	BV711	149031	0.4 μg/ml	1:500
Anti-CD38	Rt IgG2a	90	Biolegend	PE-Cy7	102718	1 μg/ml	1:200
Anti-P2RY12	Rt IgG2b	S16007D	Biolegend	PE	848004	2 μg/ml	1:100
**Intracellular**							
Anti-SYN1	Rb IgG	D12G5	Cell Signaling	AF647	11127	0.04 μg/ml	1:500
Isotype control	Rb IgG	DA1E	Cell Signaling	AF647	3452	0.04 μg/ml	1:2,500
or							
Anti-SNAP-25	Ms IgG1	SMI 81	Biolegend	AF647	836312	1 μg/ml	1:500
Isotype control	Ms IgG1	MOPC-21	Biolegend	AF647	400136	1 μg/ml	1:500

Supplementary Table 2: 12-color panel flow cytometry panel for surface staining of live microglia and BAMs followed by intracellular staining. GFP or AF488 can be used as the 12th color. Hamster (Ha), Mouse (Ms), and Rat (Rt). See Supplementary Figure 6 for gating strategy.

Antibody/dye	Species /isotype	Clone	Company	Conjuga-tion	Cat#	Conc.	Dilution
DAPI	N/A	N/A	Biolegend	None	422801	0.25 µg/ml	1:20,000
Anti-CD45	Rt IgG2b	30-F11	Thermo	PE-Cy5.5	35-0451-82	1 µg/ml	1:200
Anti-CD68	Rt IgG2a	FA-11	Biolegend	APC-Cy7	137024	0.5 µg/ml	1:400
Anti-GR1	Rt IgG2b	RB6-8C5	BD Bioscience	BUV805	741920	2 µg/ml	1:100
Anti-CD206	Rt IgG2a	C068C2	Biolegend	AF594	141712	2.5 µg/ml	1:100
Anti-CX3CR1	Ms IgG2a	SA011F11	Biolegend	BV711	149031	0.4 µg/ml	1:500
Anti-CD38	Rt IgG2a	90	Biolegend	PE-Cy7	102718	1 µg/ml	1:200
Anti-P2RY12	Rt IgG2b	S16007D	Biolegend	PE	848004	2 µg/ml	1:100
Anti-MBP*	Ms IgG1	P82H9	Biolegend	AF647	850910	2.5 µg/ml	1:200
Isotype control*	Ms IgG1	MOPC-21	Biolegend	AF647	400155	2.5 µg/ml	1:200

*Alternatively, Anti-SNAP-25 was used instead of anti-MBP (see Supplementary Table 2).

Supplementary Table 3: 9-color panel flow cytometry panel for microglia and BAMs harvested from perfusion-fixed tissue. Mouse (Ms) and Rat (Rt). See Supplementary Figure 7 for gating strategy.

FACSAria SORP II				FACSAria II		
**Lasers**	**Bandpass filters**	**Fluorophores**		**Lasers**	**Bandpass filters**	**Fluorophores**
355 nm	383/17	BUV395		405 nm	450/50	BV421
	820/60	BUV805			530/30	BV510
402 nm	450/50	BV421/DAPI			610/20	BV605
	530/30	BV510		488 nm	517/20	AF488/EGFP
	610/20	BV711		561 nm	582/15	TdTomato
488 nm	530/30	AF488/GFP*			670/14	PE-Cy5.5
	575/25	PE			780/60	PE-Cy7
	705/70	PE-Cy5.5		640 nm	661/20	AF647
	780/60	PE-Cy7			780/60	eF780
592 nm	620/20	AF594				
640 nm	670/30	AF647				
	780/60	eF780				

*The FACSAria SORP II 488 nm: 530/30 bandpass filter was also used for the detection of ZsGreen and EYFP

Supplementary Table 4: Flow cytometer configurations

Additional Supplementary Information

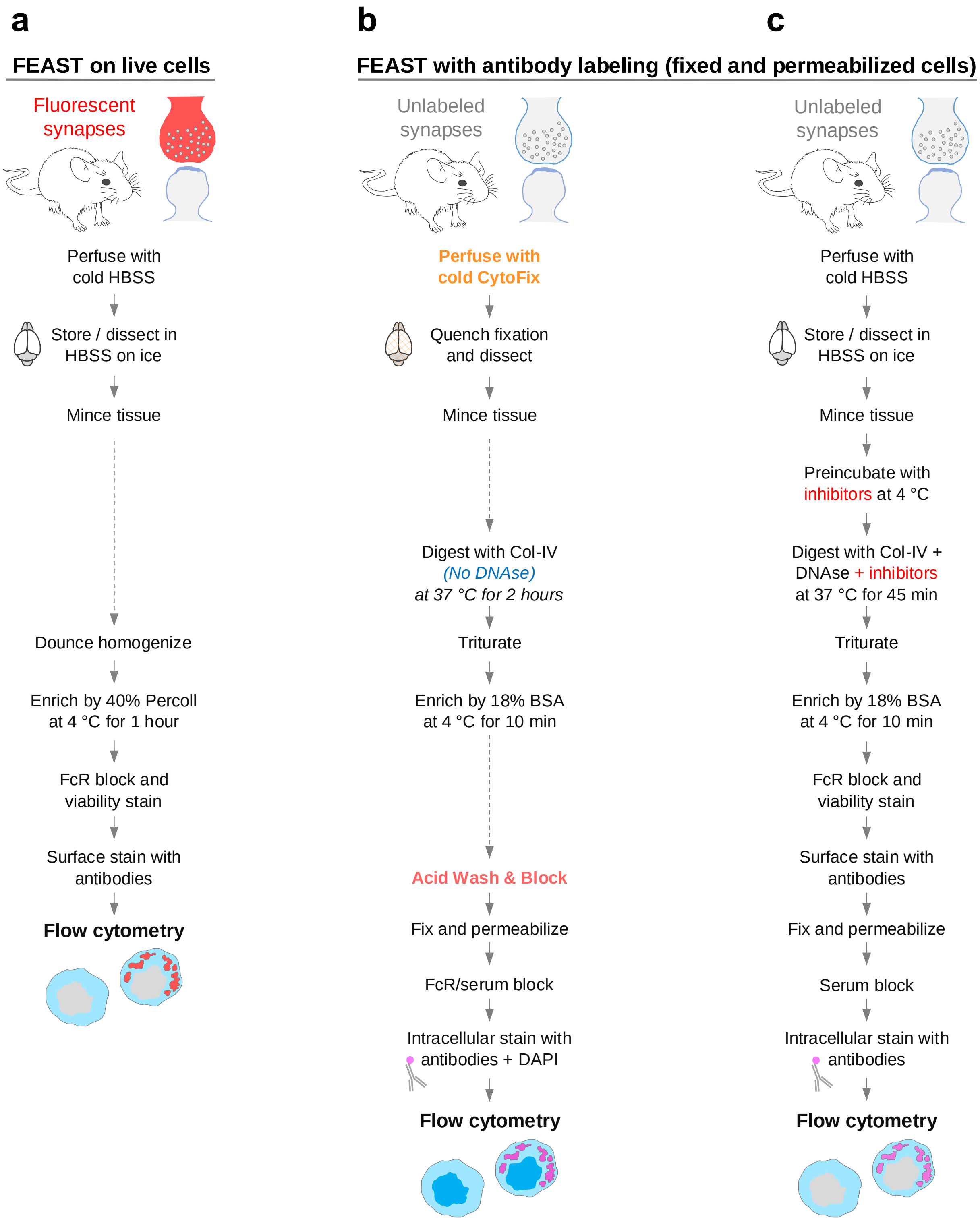

Additional Supplementary Information 1: Overview of FEAST protocols.

We propose three different strategies for interrogating in vivo engulfment depending on the specific question. Additional Supplementary Information 2, 3, and 4 provide detailed protocols with step-by-step procedures for each of these three strategies.

a) If the question is selective to microglial engulfment of in vivo labeled fluorescent material, Dounce homogenization can be used, and the microglia can be examined live. Note, Dounce homogenization does not allow for analyses of BAMs and careful attention should be paid to the pKa of the fluorescent material that is being examined. See Additional Supplementary Information 2 for details.

b) Engulfment of endogenous material by microglia and BAMs can be accomplished by harvesting the cells from perfusion-fixed tissue and subjecting them to acid wash to remove adhered debris. The engulfed material (e.g. SNAP-25 and MBP) can then be visualized by immunolabeling. See Additional Supplementary Information 3 for details.

c) Alternatively, microglial (but not BAMs) engulfment of endogenous material can be explored by introducing a cocktail engulfment and lysosomal degradation inhibitors during digestion of live tissue. This allows for the isolation and surface labeling of live microglia that can then be fixed and permeabilized, allowing the detection of engulfed material by immunolabeling. Note, mitigation of false-positive signals due to debris adherence is not part of this approach. See Additional Supplementary Information 4 for details.

FEAST on live cells isolated by cold Dounce homogenization.

Solutions to prepare ahead of time:

FACS buffer

HBSS (Life Technologies; Cat# 14175-145)
5 mM HEPES (Millipore Sigma; Cat# 83264)
0.5% BSA (Millipore Sigma; Cat# A2153)
2 mM EDTA (Fisher Scientific; Cat# 15-575-020)

90% isotonic Percoll

9 parts Percoll (Millipore Sigma; Cat# GE17-0891-01)
1 part 10x HBSS (Thermo Fisher Scientific; Cat# 14185052)

Additional equipment and solutions

Dounce homogenizer (DWK Life Sciences, Kimble; Cat# 885300-0002)
Razor blades (Fisher Scientific; Cat# 12-640)
70 µm strainer (Fisher Scientific; Cat# 22-363-548)
FcR block (anti-CD16/CD32), viability dye, and antibodies for surface staining (Supplementary Table 2)
Brilliant Stain Buffer Plus (BD; Cat# 566385)
96-well U-bottom polypropylene plate (Thermo Fisher Scientific; Cat# 267334)
Polystyrene tubes with 35 µm strainer cap (Corning Life Science; Cat# 352235)

Good practices:

● Keep all solutions cold when working with live cells: Store HBSS and pre-made solutions at 4 °C or on ice and pre-cool centrifuges to 4 °C. Keep all tubes, Dounce homogenizers, and plates containing tissue or cells on ice or in the fridge to minimize cell death and artifactual ex vivo engulfment.
● Avoid generating bubbles when pipetting and when Dounce homogenizing.
● Cover fluorescent samples with foil.

Step-By-Step procedures (all steps are performed on ice):

1) Fully anesthetize the mouse and transcardially perfuse with ice-cold HBSS.
Perfuse with ~10 times the estimated blood volume (e.g. 20 ml HBSS for a 20 g mouse).
2) Remove the brain and dissect the region(s) of interest.
3) Mince the tissue with razor blades until it resembles a fine paste. *If the dissected tissue is very small (e.g. an LGN), transfer it to a 2 ml tube and chop in 1 ml ice-cold FACS buffer with scissors (roughly 200 times) instead of using razor blades.*
4) Transfer the minced tissue to a 2 ml Dounce homogenizer in 1 ml ice-cold FACS buffer. Gently homogenize by completing 20-30 strokes with pestle 'A' followed by 25-30 strokes with pestle 'B'.
5) Filter the homogenate into a 15 ml tube (pre-coated with FACS buffer) through a 70 µm strainer.
6) Fill up to 5.5 ml with ice cold FACS buffer and add 4.5 ml of ice-cold 90% isotonic Percoll. Cap and invert 10 times to mix. *This creates 10 ml of 40% Percoll.*
7) Enrich for microglia by centrifuging for 1 hour at 500 x g, 4 °C.
8) Remove the supernatant* and resuspend the pellet in 2 ml of ice-cold FACS buffer.
**Suck off the top myelin layer and most of the liquid volume using a vacuum pump. Remove the last 1 ml with a P1000 pipette.*
9) Transfer each sample to a 2 ml round bottom tube and spin at 500 x g for 8 min, 4 °C. *This step removes residual Percoll.*
10) Decant the supernatant and resuspend in 150 µl of ice-cold FACS buffer.
11) Transfer the cells to a 96 well U-bottom plate, spin at 500 x g for 5 min at 4 °C and decant the supernatant.
Decanting the supernatant from the plate is done by flipping the plate upside down in one swift motion.
12) Incubate in 50 µl of HBSS + 2 mM EDTA with FcR block and fixable viability dye for 20 min on ice.
13) Add 50 µl of FACS buffer containing the mix of antibodies against desired surface markers* and incubate for 20 min on ice. **Add 20% Brilliant Stain Buffer Plus if brilliant dyes are part of the panel. Also, remember to double the desired final concentration of antibodies added here to account for the extra volume from step 12.*
14) Add 100 µl of FACS buffer and spin at 500 x g for 5 min at 4 °C and decant the supernatant.
15) Wash in 200 µl ice-cold FACS buffer, spin at 500 x g for 5 min at 4 °C, and decant the supernatant.
16) Resuspend in ice-cold FACS buffer and filter the samples through a 35 µm strainer. *We have found that 300-400 µl total volume is ideal for analyzing cells from up to half of a brain.*
The samples are ready for flow cytometric analysis.

Additional Supplementary Information 2: Detailed protocol for FEAST on live cells isolated by cold Dounce homogenization.

FEAST on cells harvested from perfusion fixed tissue

Solutions to prepare ahead of time:

FACS buffer
HBSS (Life Technologies; Cat# 14175-145)
5 mM HEPES (Millipore Sigma; Cat# 83264)
0.5% BSA (Millipore Sigma; Cat# A2153)
2mM EDTA (Fisher Scientific; Cat# 15-575-020)

25% BSA gradient
BSA (25% w/v)
HBSS

Digestion buffer (RPMI-H)
RPMI-1640 without phenol red (Fisher Scientific; Cat# 11835-055)
5 mM HEPES

Digestion mix: (2 mL/brain region or 4 mL per brain): *pre-heat to 37 °C*.
RPMI-H
800 U/mL Collagenase IV (Worthington; Cat# LS004189)
No DNAse.

PFA quenching solution (GnT)
HBSS
5 mM HEPES
250 mM Tris (Millipore Sigma; Cat# GE17-1321-01)
250 mM Glycine (Millipore Sigma; Cat# G7126)

Acid wash solution (~pH 2.75)**:** *pre-cool to 4 °C before using*.
0.9% saline (Millipore Sigma, Cat# S8776)
0.2 M Acetic acid (Millipore Sigma, Cat# A6283)

Blocking solution:
HBSS
5 mM HEPES
20% Ovalbumin (Millipore Sigma, Cat# A5503) *Dissolve in cold HBSS by stirring in small quantities at a time.*
20% Mouse serum (Millipore Sigma, Cat# M5905)

Additional equipment and solutions

Fixation buffer (BD; Cat# BD 550010 or Biolegend Cat# 420801)
Razor blades (Fisher Scientific; Cat# 12-640)
70 µm strainer (Fisher Scientific; Cat# 22-363-548)
96-well U-bottom polypropylene plate (Thermo Fisher Scientific; Cat# 267334)
Permeabilization buffer (Thermo Fisher Scientific; Cat# 00833356)
Goat serum (Millipore Sigma; Cat# G9023)
FcR block (anti-CD16/CD32), DAPI, and antibodies (Supplementary Table 3)
Brilliant Stain Buffer Plus (BD; Cat# 566385)
Polystyrene tubes with 35 µm strainer cap (Corning Life Science; Cat# 352235)

Good practices:

- Avoid generating bubbles when pipetting and triturating.
- Cover fluorescent samples with foil.

Step-By-Step procedures

1) Fully anesthetize the mouse and transcardially perfuse with 10 ml of ice-cold HBSS.
2) Continue perfusion with 20 ml of ice-cold fixation buffer at a flow rate of 7 ml/min.
3) Remove the brain and submerge it in GnT solution at room temperature (RT). *This quenches residual PFA. The remaining steps can be performed at RT.*
4) Dissect brain region(s) of interest and mince the tissue with razor blades until it resembles a fine paste. *If the dissected tissue is very small (i.e. an LGN), transfer it to a 2 ml tube and chop in 1 ml GnT solution with scissors (roughly 200 times).*
5) Transfer the minced tissue to a 2 (or 4) ml tube with GnT and spin at 300 x g for 3 min.
6) Decant the supernatant and resuspend the pellet in 2 ml (or 4 ml) of pre-heated digestion mix.
7) Incubate for 2 hours at 37°C while gently agitating.
8) Spin at 500 x g for 5 min and decant the supernatant.
9) Add 1 ml FACS buffer to stop the digestion and triturate with a P1000 pipette.
Triturate 20 times lightly followed by 20 times with more force or until fully homogenized.
10) Filter the homogenate into a 15 ml tube (pre-coated with FACS buffer) through a 70 µm filter.
11) Adjust the volume to 2 ml with FACS buffer and add 5 mL of 25% BSA. Invert 10 times to mix.
12) Spin at 1200 x g for 10 min. *We find that this BSA gradient works better than Percoll to enrich for microglia and BAMs when paired with this isolation protocol.*
13) Remove the supernatant* and resuspend the pellet in 2 ml of FACS buffer.
**Suck off the top myelin layer and most of the liquid volume using a vacuum pump. Remove the last 1 ml with a P1000 pipette.*
14) Transfer each sample to a 2 ml round bottom tube and spin at 500 x g for 8 min. *This step removes residual BSA.*
15) Decant the supernatant and resuspend in 150 µl FACS buffer.
16) Transfer the cells to a 96 well U-bottom plate, spin at 500 x g for 5 min, and decant the supernatant.
Decanting the supernatant from the plate is done by flipping the plate upside down in one swift motion.
17) Resuspend the pellet in 200 µl of cold acid wash solution and incubate for 15 min on ice.
18) Spin at 500 x g for 5 min at 4 °C and decant the supernatant.
19) Resuspend the pellet in 200 µl blocking solution and incubate for 20 m.
20) Spin at 800 x g for 8 min and decant the supernatant.
21) Fix the cells in 200 µl fixation buffer for 20 mi.
This additional fixation step is necessary for the cells to last permeabilization and storage.
22) Spin at 500 x g for 5 min, discard the supernatant, and resuspend the pellet in 150 µl FACS buffer.
At this point, the sample can be washed once and stored in the fridge in FACS buffer, and the staining continued at a later time.
23) Split the samples for the isotype control staining.
24) Add 200 µl permeabilization buffer, spin at 500 x g for 5 min, and decant the supernatant.
25) Add 50 µl of permeabilization buffer with FcR Block and 10% goat serum and incubate for 20 min.
26) Add 50 µl permeabilization buffer + DAPI and antibody cocktail* and incubate for 20 mi.
**Add 20% Brilliant Stain Buffer Plus if brilliant dyes are part of the panel. Also, remember to double the desired final concentration of DAPI and antibodies added here to account for the extra volume from step 25.*
27) Wash in 200 µl permeabilization buffer, spin at 500 x g for 5 min, and decant the supernatant.
28) Wash in 200 µl FACS buffer, spin one last time at 500 x g for 5 min, and decant the supernatant.
29) Resuspend in FACS buffer and filter the samples through a 35 µm strainer. *We have found that 300-400 µl total volume is ideal for analyzing cells from up to half of a brain.*
The samples are ready for flow cytometric analysis.

Additional Supplementary Information 3: Detailed protocol for FEAST on cells harvested from perfusion-fixed tissue.

FEAST on cells isolated by enzymatic digestion with inhibitor cocktail

Solutions to prepare ahead of time:

FACS buffer
HBSS (Life Technologies; Cat# 14175-145)
5 mM HEPES (Millipore Sigma; Cat# 83264)
0.5% BSA (Millipore Sigma; Cat# A2153)
2mM EDTA (Fisher Scientific; Cat# 15-575-020)

25% BSA gradient
BSA (25% w/v)
HBSS

Digestion buffer (RPMI-H)
RPMI-1640 without phenol red (Fisher Scientific; Cat# 11835-055)
5 mM HEPES

Digestion mix: (2 mL/brain region or 4 mL per brain): *pre-heat to 37 °C.*
RPMI-H
800U/mL Collagenase IV (Worthington; Cat# LS004189)
250 U/mL DNAse-1 (Worthington; Cat# LK003172)
Inhibitor cocktail (diluted 1:1000 from stock solution)

Inhibitor cocktail (1,000^{x} stock solution):
2 mM Cytochalasin D (Tocris; Cat# 1233)
2 mM Wortmannin (Tocris; Cat# 1232)
25 mM Pitstop 2 (Abcam; Cat# ab120687)
40 mM Dynasore (Tocris; Cat# 2897)
40 µM Bafilomycin A1 (Tocris; Cat# 1334)

Additional equipment and solutions
Razor blades (Fisher Scientific; Cat# 12-640)
70 µm strainer (Fisher Scientific; Cat# 22-363-548)
96-well U-bottom polypropylene plate (Thermo Fisher Scientific; Cat# 267334)
FcR block (anti-CD16/CD32), DAPI, and antibodies (Supplementary Table 2)
Brilliant Stain Buffer Plus (BD; Cat# 566385)
Fixation buffer (BD; Cat# BD 550010 or Biolegend Cat# 420801)
Permeabilization buffer (Thermo Fisher Scientific; Cat# 00833356)
Goat serum (Millipore Sigma; Cat# G9023)
Polystyrene tubes with 35 µm strainer cap (Corning Life Science; Cat# 352235)

Good practices:
- Keep all solutions ice-cold when working with live cells without the inhibitor cocktail (Step 1-7 and 9-18): Store HBSS and pre-made solutions at 4 °C or on ice and pre-cool centrifuges at 4 °C. Keep all tubes, and plates containing tissue or cells on ice or in the fridge to minimize cell death and artifactual ex vivo engulfment.
- Avoid generating bubbles when pipetting and triturating
- Cover fluorescent samples with foil.

Step-By-Step procedures

1) Fully anesthetize the mouse and transcardially perfuse with ice-cold HBSS.
Perfuse with ~10 times the estimated blood volume (e.g. 20 ml HBSS for a 20 g mouse).
2) Remove the brain and dissect the brain region(s) of interest in ice-cold HBSS.
3) Mince the tissue with razor blades until it resembles a fine paste. *If the dissected tissue is very small (e.g. an LGN) transfer it to a 2 ml tube and chop in 1 ml ice-cold HBSS with scissors (roughly 200 times) instead of using razor blades.*
4) Transfer the minced tissue to a 2 (or 4 ml) tube with ice-cold RPMI-H and spin at 300 x g for 3 min.
5) Decant the supernatant and resuspend the pellet in 2 ml (or 4 ml) of ice-cold RPMI-H + inhibitor cocktail
6) Incubate for 20 min at 4°C while gently agitating. *This step allows pretreatment with the inhibitors before the digest.*
7) Spin at 300 x g for 3 min, decant the supernatant, and resuspend the pellet in pre-heated digestion mix containing the inhibitor cocktail.
8) Incubate for 45 min at 37°C while gently agitating.
9) Spin at 500 x g for 5 min at 4°C and discard the supernatant.
10) Add 1 ml of ice-cold FACS buffer to stop the digestion and triturate with a P1000 pipette.
Triturate 10-20 times lightly followed by 20-30 times with more force or until fully homogenized.
11) Filter the homogenate into a 15 ml tube (pre-coated with FACS buffer) through a 70 µm filter.
12) Adjust the volume to 2 ml with FACS buffer and add 5 ml of ice-cold 25% BSA. Invert 10 times to mix.
13) Spin at 1200 x g for 10 min. *We find that this BSA gradient works better than Percoll to enrich for microglia and BAMs when paired with this isolation protocol.*
14) Remove the supernatant* and resuspend the pellet in 2 ml of ice-cold FACS buffer.
**Suck off the top myelin layer and most of the liquid volume using a vacuum pump. Remove the last 1 ml with a P1000 pipette.*
15) Transfer each sample to a 2 ml round bottom tube and spin at 500 x g for 8 min. *This step removes residual BSA.*
16) Decant the supernatant and resuspend in 150 µl ice-cold FACS buffer.
17) Transfer the cells to a 96 well U-bottom plate, spin at 500 x g for 5 min at 4°C and decant the supernatant.
Decanting the supernatant from the plate is done by flipping the plate upside down in one swift motion.
18) Incubate cells in 50 µl of ice-cold HBSS + 2 mM EDTA with FcR Block and fixable viability dye for 20 min on ice.
19) Add 50 µl of ice-cold FACS buffer containing the mix of antibodies against desired surface markers* and incubate for 20 min on ice.
**Add 20% Brilliant Stain Buffer Plus if brilliant dyes are part of the panel. Also, remember to double the desired final concentration of DAPI and antibodies added here to account for the extra volume from step 18.*
20) Add 100 µl of ice-cold FACS buffer and spin at 500 x g for 5 min at 4 °C and decant the supernatant.
21) Wash in 200 µl ice-cold FACS buffer, spin at 500g for 5 min at 4 °C and decant the supernatant.
22) Fix in 200 µl fixation buffer for 20 min at room temperature (RT).
23) Spin at 500 x g for 5 min, discard the supernatant, and resuspend the pellet in 150 µl FACS buffer.
At this point, the sample can be washed once and stored in the fridge in FACS buffer, and the staining continued at a later time.
24) Split the samples for the isotype control staining.
The remaining steps can be performed at RT.
25) Add 200 µl permeabilization buffer and spin at 500 x g for 5 min.
26) Add 50 µl permeabilization buffer with 10% goat serum and incubate for 20 min.
27) Add 50 µl permeabilization buffer with antibodies for intracellular staining and incubate for 20 min.
28) Wash in 200 µl permeabilization buffer, spin at 500 x g for 5 min, and decant the supernatant.
29) Wash in 200 µl FACS buffer, spin one last time at 500 x g for 5 min, and decant the supernatant.
30) Resuspend in FACS buffer and filter the samples through a 35 µm strainer. *We have found that 300-400 µl total volume is ideal for analyzing cells from up to half of a brain.*
The samples are ready for flow cytometric analysis.

Additional Supplementary Information 4: Detailed protocol for FEAST on cells isolated by enzymatic digestion with inhibitor cocktail.
