# Supplementary material for: FEAST: A flow cytometry-based toolkit for interrogating microglial engulfment of synaptic and myelin proteins
Source: Nat Commun. 2023 Sep 27;14:6015. doi: 10.1038/s41467-023-41448-7 (PMC10533836; doi:10.1038/s41467-023-41448-7)
Supplement: Supplementary file 3 — Reporting Summary [file 41467_2023_41448_MOESM3_ESM.pdf]

nature portfolio

Corresponding author(s): Beth Stevens

Last updated by author(s): Aug 18, 2023

Reporting Summary

Nature Portfolio wishes to improve the reproducibility of the work that we publish. This form provides structure for consistency and transparency in reporting. For further information on Nature Portfolio policies, see our Editorial Policies and the Editorial Policy Checklist.

Statistics

For all statistical analyses, confirm that the following items are present in the figure legend, table legend, main text, or Methods section.

n/a	Confirmed	
☐	☒	The exact sample size (*n*) for each experimental group/condition, given as a discrete number and unit of measurement
☐	☒	A statement on whether measurements were taken from distinct samples or whether the same sample was measured repeatedly
☐	☒	The statistical test(s) used AND whether they are one- or two-sided<br>*Only common tests should be described solely by name; describe more complex techniques in the Methods section.*
☒	☐	A description of all covariates tested
☐	☒	A description of any assumptions or corrections, such as tests of normality and adjustment for multiple comparisons
☐	☒	A full description of the statistical parameters including central tendency (e.g. means) or other basic estimates (e.g. regression coefficient) AND variation (e.g. standard deviation) or associated estimates of uncertainty (e.g. confidence intervals)
☐	☒	For null hypothesis testing, the test statistic (e.g. *F*, *t*, *r*) with confidence intervals, effect sizes, degrees of freedom and *P* value noted<br>*Give P values as exact values whenever suitable.*
☒	☐	For Bayesian analysis, information on the choice of priors and Markov chain Monte Carlo settings
☒	☐	For hierarchical and complex designs, identification of the appropriate level for tests and full reporting of outcomes
☒	☐	Estimates of effect sizes (e.g. Cohen's *d*, Pearson's *r*), indicating how they were calculated

Our web collection on statistics for biologists contains articles on many of the points above.

Software and code

Policy information about availability of computer code

Data collection	Flow cytometric data were collected using BD FACSDIVA version 8.0.1 software and microscopy images were collected using Zeiss Zen Black v2.3 software
Data analysis	FlowJo_v10.8.0 software was used to analyze flow cytometric data and Fiji/ImageJ 2.3.1 were used for the processing of microscopy images

For manuscripts utilizing custom algorithms or software that are central to the research but not yet described in published literature, software must be made available to editors and reviewers. We strongly encourage code deposition in a community repository (e.g. GitHub). See the Nature Portfolio guidelines for submitting code & software for further information.

Data

Policy information about availability of data

All manuscripts must include a data availability statement. This statement should provide the following information, where applicable:

- Accession codes, unique identifiers, or web links for publicly available datasets
- A description of any restrictions on data availability
- For clinical datasets or third party data, please ensure that the statement adheres to our policy

The authors confirm that all relevant data are included in the paper and that none of the data generated during this study has been removed. Source data are provided with this paper and data further supporting the findings of this study are available upon request.

Human research participants

Policy information about studies involving human research participants and Sex and Gender in Research.

Reporting on sex and gender	N/A
Population characteristics	N/A
Recruitment	N/A
Ethics oversight	N/A

Note that full information on the approval of the study protocol must also be provided in the manuscript.

Field-specific reporting

Please select the one below that is the best fit for your research. If you are not sure, read the appropriate sections before making your selection.

☒ Life sciences ☐ Behavioural & social sciences ☐ Ecological, evolutionary & environmental sciences

For a reference copy of the document with all sections, see nature.com/documents/nr-reporting-summary-flat.pdf

Life sciences study design

All studies must disclose on these points even when the disclosure is negative.

Sample size	The sample sizes were predetermined for each experiment and no samples were added or removed. n=4 or n=5 were chosen as the standard sample size in this study to ensure a thorough assessment of the biology in question. The experimental manipulations used in this study (in vitro: treatment with synaptosomes versus no treatment or treatment with myelin versus no treatment and in vivo: optic nerve crush versus no crush, injection of lysolecithin versus no injections, treatment with cuprizone versus control diet) were designed to provide strong effect for clear comparisons and the chosen sample size were predicted to be sufficient to address the biological questions.<br>n-values for each experiment are provided in the figure legends and additional details for each sample are provided in the source data.
Data exclusions	No data were excluded from the analysis of each experiment.
Replication	Figure 1a: Representative image of hundreds of microglia examined. Repeated multiple times<br>Figure 1b: Representative image of hundreds of microglia examined. One experiment<br>Figure 1d-e: n=4 mice per condition. Conducted in two rounds, n=2 mice per round<br>Figure 2b: n=5 for optic nerve crush and n=4 for no crush. Conducted as one experiment<br>Figure 2c: n=8 for optic nerve crush and n=5 for no crush. Conducted in two rounds, n=5 for optic nerve crush and n=3 for no crush in first round and n=3 for optic nerve crush and n=2 for no crush in second round.<br>Figure 2d: n=5 for optic nerve crush and n=5 for no crush. Conducted as one experiment<br>Figure 2e: n=5 for optic nerve crush and n=4 for no crush. Conducted as one experiment<br>Figure 3b: Representative image of tens of synaptosomes examined. One experiment<br>Figure 3c. The n-value represents the number of independent replications comprising an individual batch of EOC20 and new batch of synaptosomes. n=4 for all antibodies except SNAP-25 MS, SYN1/2 Ck, GAS65 Gp, Homer 1b/c Rb, GluA1 Rb (2), and IgG1 Ms for which n=3.<br>Figure 3e. The n-value represents the number of independent replications comprinsing an individual batch of EOC20 and new batch of myelin). n=4 for all antibodies<br>Figure 4d-e. n=4 mice per condition. Conducted in two rounds with n=2 mice per condition in each round<br>Figure 5b-c. n=4 mice per condition. One experiment<br>Figure 6b-e. n=5 mice per condition. One experiment<br>Figure 7b-c. n=4 mice per condition. Conducted in two rounds with n=2 mice per condition n each round<br>Figure 7e-f. n=4 mice per condition. One experiment
Randomization	Cells and mice were randomly assigned to experimental groups.
Blinding	The experimenter(s) were not blinded to the experimental groups. Alternating between the experimental conditions during sample collection, preparation, and analysis was prioritied as more practically feasible.

Reporting for specific materials, systems and methods

We require information from authors about some types of materials, experimental systems and methods used in many studies. Here, indicate whether each material, system or method listed is relevant to your study. If you are not sure if a list item applies to your research, read the appropriate section before selecting a response.

Materials & experimental systems

n/a	Involved in the study	
☐	☒	Antibodies
☐	☒	Eukaryotic cell lines
☒	☐	Palaeontology and archaeology
☐	☒	Animals and other organisms
☒	☐	Clinical data
☒	☐	Dual use research of concern

Methods

n/a	Involved in the study	
☒	☐	ChIP-seq
☐	☒	Flow cytometry
☒	☐	MRI-based neuroimaging

Antibodies

Antibodies used

Three tables (Supplementary Tables 1-3) are provided with the name, species, isotype, clone, company, catalog number, and the concentration used for each of the antibodies used in this study.

Validation

All primary antibodies were initially used at dilutions recommended for flow cytometry by the manufacturer.
Antibodies against synaptic proteins were validated on cells that had engulfed synaptic material (results are displayed in Figure 3c and antibody details are provided Supplementary Table 1).
Antibodies against myelin basic protein were validated on cells that had engulfed myelin (results are displayed in Figure 3e).
Common antibodies for flow cytometric identification of specific cellular markers were used in accordance with the manufacturer's website and the specific concentrations used in our study are provided in Supplementary Table 2 and 3.

Eukaryotic cell lines

Policy information about cell lines and Sex and Gender in Research

Cell line source(s)

EOC20 cells (ATCC; CAT# CRL-2469). This immortalized cell line is derived from a female C3H/HeJ mouse according to manufacturer.

Authentication

The cells were ordered directly from ATCC and were not authenticated at Boston Children's Hospital.

Mycoplasma contamination

The cell line was not tested for mycoplasma contamination

Commonly misidentified lines
(See ICLAC register)

No commonly misidentified lines were used in this study

Animals and other research organisms

Policy information about studies involving animals; ARRIVE guidelines recommended for reporting animal research, and Sex and Gender in Research

Laboratory animals

C57BL/6J mice (JAX stock No: 000664), used for experiments at P56-P130 and P18-P21 for generation of primary microglial cultures
C57BL/6NJ (JAX stock No: 005304), used at experiments at P90

CHX10-Cre (Tg(Chx10-EGFP/cre,-ALPP)2Clc/J: JAX Stock No: 005105), used for breeding
lsl:ZsGreen (B6.Cg-Gt(ROSA)26Sortm6(CAG-ZsGreen1)Hze/J: JAX Stock No: 007906), used for breeding
lsl:TdTomato (B6.Cg-Gt(ROSA)26Sortm9(CAG-tdTomato)Hze/J: JAX Stock No: 007909), used for breeding
lsl:EGFP (B6.129(Cg)-Gt(ROSA)26Sortm4(ACTB-tdTomato,-EGFP)Luo/J: JAX Stock No: 007676), used for breeding
lsl:EYFP (B6.129X1-Gt(ROSA)26Sortm1(EYFP)Cos/J: JAX Stock No: 006148), used for breeding
SYN1-KO mice (B6.129P2-Syn1tm1Pggd/Mmja), JAX Stock No: 41436-JAX), used for breeding
Ubi-GFP (C57BL/6-Tg(UBC-GFP)30Scha/J) JAX Stock No: 004353), used for breeding

CHX10-Cre x lsl:ZsGreen, used for experiments at P75-83
CHX10-Cre x lsl:TdTomato, used for experiments at P120-340
CHX10-Cre x lsl:EGFP, used for experiments at P140-200
CHX10-Cre x lsl:EYFP, used for experiments at P76-78
SYN1-KO:Ubi-GFP, used for experiments at P56-70

Wild animals

No wild animals were used in this study

Reporting on sex

The sex of the experimental animals used in this study is stated in the figure legends. Male and female mice were balanced, for each experimental design, as much as practically feasible.

Field-collected samples

No field-collected samples were used in this study

Ethics oversight

All housing and experimental procedures were approved and overseen by Boston Children’s Hospital Institutional Animal Care and Use Committee following NIH guidelines for the humane treatment of animals.

Note that full information on the approval of the study protocol must also be provided in the manuscript.

Flow Cytometry

Plots

Confirm that:

☒ The axis labels state the marker and fluorochrome used (e.g. CD4-FITC).

☒ The axis scales are clearly visible. Include numbers along axes only for bottom left plot of group (a 'group' is an analysis of identical markers).

☐ All plots are contour plots with outliers or pseudocolor plots.

☒ A numerical value for number of cells or percentage (with statistics) is provided.

Methodology

Sample preparation	Detailed descriptions of sample preparation is included in the Method section of the manuscript and in the supplementary information.<br>Single cell suspensions were generated from mouse brains. The mice were perfused, the brains isolated, and single cell homogenates were generated by mechanical dissociation (Dounce homogenizations or titration following enzymatic digestion). Microglia and brain-associated macrophages where enriched by centrifugation in Percoll or bovine serum albumin.<br>Primary microglial cells for culturing (supplementary figure 1) were harvested from the brain of WT mice. The brains were Dounce homogenized and microglia were enriched by Percoll centrifugation.
Instrument	Data were collected using a FACSAria II or a FACSAria SORP II.<br>Supplementary Table 4 provides an overview of the flow cytometers used in this study with information about the specific lasers and bandpass filters.
Software	Flow cytometric data were collected using BD FACSDIVA version 8.0.1 and analyzed using FlowJo_v10.8.0 software.
Cell population abundance	Cell numbers are provided in the figure legends. Flow cytometry was used for analyses. No cell sorting was conducted.
Gating strategy	The overall gating strategy for individual experiments is stated in the main figure legends. Full gating strategies including 'FMO' (fluorescence minus one) controls are displayed in supplementary data figure 6 and 7 with pseudocolor plots and axis labels stating the marker and fluorochrome used. Representative gating strategies for figure 1 and 2 are provide in supplementary figure 8.

☒ Tick this box to confirm that a figure exemplifying the gating strategy is provided in the Supplementary Information.
